# The Bone Dysplasia Ontology: integrating genotype and phenotype information in the skeletal dysplasia domain

**DOI:** 10.1186/1471-2105-13-50

**Published:** 2012-03-26

**Authors:** Tudor Groza, Jane Hunter, Andreas Zankl

**Affiliations:** 1 School of ITEE, The University of Queensland, St. Lucia, Australia; 2 Bone Dysplasia Research Group, UQ Centre for Clinical Research (UQCCR), The University of Queensland, Herston, Australia; 3 Genetic Health Queensland, Royal Brisbane and Women's Hospital, Herston, Australia

## Abstract

**Background:**

Skeletal dysplasias are a rare and heterogeneous group of genetic disorders affecting skeletal development. Patients with skeletal dysplasias suffer from many complex medical issues including degenerative joint disease and neurological complications. Because the data and expertise associated with this field is both sparse and disparate, significant benefits will potentially accrue from the availability of an ontology that provides a shared conceptualisation of the domain knowledge and enables data integration, cross-referencing and advanced reasoning across the relevant but distributed data sources.

**Results:**

We introduce the design considerations and implementation details of the Bone Dysplasia Ontology. We also describe the different components of the ontology, including a comprehensive and formal representation of the skeletal dysplasia domain as well as the related genotypes and phenotypes. We then briefly describe SKELETOME, a community-driven knowledge curation platform that is underpinned by the Bone Dysplasia Ontology. SKELETOME enables domain experts to use, refine and extend and apply the ontology without any prior ontology engineering experience--to advance the body of knowledge in the skeletal dysplasia field.

**Conclusions:**

The Bone Dysplasia Ontology represents the most comprehensive structured knowledge source for the skeletal dysplasias domain. It provides the means for integrating and annotating clinical and research data, not only at the generic domain knowledge level, but also at the level of individual patient case studies. It enables links between individual cases and publicly available genotype and phenotype resources based on a community-driven curation process that ensures a shared conceptualisation of the domain knowledge and its continuous incremental evolution.

## Background

Skeletal dysplasias are a heterogeneous group of genetic disorders affecting skeletal development. There are currently over 450 recognised types, clustered in 40 groups. Patients with skeletal dysplasias have complex medical issues including short stature, degenerative joint disease, scoliosis and neurological complications. These patients are also a precious resource for biomedical research as they enable scientists to study the effects of single genes on human bone and cartilage development and function. The resulting insights lead to a better understanding of the pathogenesis of more common connective tissue disorders such as arthritis or osteoporosis.

Despite their importance, bone dysplasias are not exploited to their full potential in biomedical research. Since most conditions are rare (< 1:10'000 births) and correct diagnosis is difficult, only a few medical centres worldwide have expertise in diagnosis and management of these disorders. On the other hand, the identification of many skeletal dysplasia genes and subsequent studies of their functions and interactions have led to an explosion of knowledge about bone and cartilage biology. The biomedical literature now contains a large amount of information about individual genes and gene interactions [1], but it is often difficult to grasp how these interactions work together in a broader context, such as at the growth plate. In turn, the focus on specific cases or genes makes it difficult to identify etiological relationships between skeletal dysplasias, or to recognise clinical or radiological characteristics that are indicative of defects associated with specific molecular pathways.

The International Skeletal Dysplasia Society http://www.isds.ch/ has attempted to address some of these problems with its Nosology of Genetic Skeletal Disorders. Since 1972, the ISDS Nosology lists all recognised skeletal dysplasias and groups them by common clinical-radiographic characteristics and/or molecular disease mechanisms. The ISDS Nosology is revised every 4 years by an expert committee and the updated version is published in a medical journal. The latest version is from 2010 and is presented in [2]. The ISDS Nosology is widely accepted as the "official" nomenclature for skeletal dysplasias within the biomedical community.

While the content of the Nosology is invaluable, the format of the Nosology has several shortcomings. Firstly, the classification scheme is inflexible, each disorder is listed in one group, based either on its clinical radiographic appearance or on its underlying molecular genetic mechanism (many disorders can be associated with multiple groups). Secondly, very limited information is listed for each entry. Current information is limited to: the OMIM [3] number, the chromosome locus, gene name and protein name. In other words, the Nosology is not linked to freely available and widely used online repositories such as UniProt [4], limiting users' ability to further study the disorders. Thirdly, the Nosology associates diseases with specific genes but provides no additional information on the responsible gene mutations. Fourthly, phenotypic and clinical-radiographic information is present intrinsically in the classification, but not explicitly in the Nosology. Finally, due to its current publishing process, the content quickly becomes outdated, as genes or disorders discovered after the publication date cannot be included until the next revision (4 years later). For example, shortly after the publication of the newest version of the ISDS Nosology, Gray et al. [5] have shown that the Serpentine fibula polycystic kidney syndrome (SFPKS) is characterised by truncating mutations in NOTCH2, and consequently have proposed the move of SFPKS from the Filamin Group to the Osteolysis Group, due to its genetic similarities with the Hajdu-Cheney syndrome. Unfortunately, this information will be reflected in the Nosology only in four years time.

Over the past 10 years, ontologies have proven to represent a practical solution to data integration and knowledge acquisition, processing and management, particularly in the Healthcare and Life Sciences [6]. Their use in automated annotation [7,8] or cross-linking for query and retrieval purposes [9,10] is now broadly recognised in the biomedical field. As a result of their wide adoption and in order to enable collaboration and cross-fertilisation, several ontology repositories and collections have been created. The Open Biomedical Ontologies Foundry http://www.obofoundry.org/[11] represents the most prominent collaborative collection of biomedical ontologies, while the NCBO BioPortal http://bioportal.bioontology.org/[12] is currently the most comprehensive ontology repository in this domain. Ontologies hosted in or linked from these two access points vary widely in size (ranging from several hundreds to hundreds of thousands of concepts) and domain (from imaging methods to cell behaviour or clinical terminology). While an extensive number of biomedical topics have been covered, there remain topics where more comprehensive documentation is required. One such topic is the skeletal dysplasia domain.

The Bone Dysplasia Ontology aims to complement the spectrum of existing ontologies and address the specific knowledge representation shortcomings of the ISDS Nosology. Its main role is to provide the scaffolding required for a comprehensive, accurate and formal representation of the genotypes and phenotypes involved in skeletal dysplasias, together with their specific and disease-oriented constraints. As opposed to the current ISDS Nosology, the ontology enables a shared conceptual model, formalised in a machine-understandable description, in addition to a continuous evolution and a foundational building block for facilitating knowledge extraction and reasoning. The symbiosis between the ontology and the community-driven knowledge curation platform built to support its evolution enables collaborative and incremental acquisition and encoding of advances by the experts in the field. Ultimately, it underpins mechanisms for sharing and re-use of data and information and advanced reasoning techniques for semi-automated diagnosis or disease features extraction.

## Methods

The Bone Dysplasia Ontology has been built collaboratively by a team of experts in skeletal dysplasias and ontology engineering. The design of the ontology was heavily influenced by the need to address the limitations of the ISDS Nosology, or more concretely, the need to capture the wealth of intrinsic knowledge of the domain described in diverse case studies or publications. Hence, the main purpose of the ontology coincides with its implicit role of providing a shared conceptualisation of the domain, and is not necessarily dependent on specific use cases. The community-driven knowledge curation platform built to support the ontology (described later in the article) enables a knowledge engineering cycle that combines a sustainable ontology evolution and quality-oriented process (enforced by the editorial roles embraced by the experts in the community) with the direct use of the ontology for semantic annotation of clinical summaries and collaborative decision-making.

**Figure 1:**
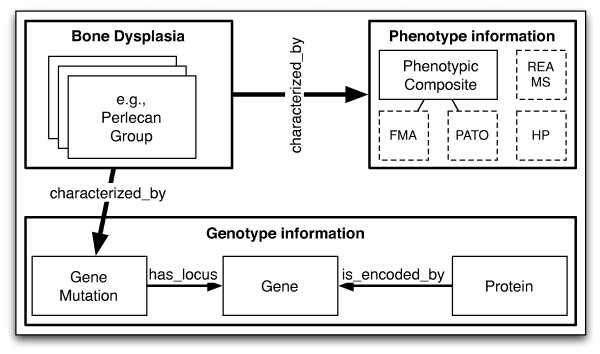
**The Bone Dysplasia Ontology**. Generic overview of the Bone Dysplasia ontology structure. The high level boxes represent the three pillars of the domain, i.e., bone dysplasias, phenotype and genotype information. The dotted boxes in the Phenotype information pillar denote relationships-based dependencies on particular concepts from those ontologies and not subsumption.

The aim of the ontology is to support the evolving knowledge in the skeletal dysplasia domain by providing a formal foundation to be used by the community to continuously update the classification of the disease concepts, thus improving to the current publishing cycle practice. A second, yet equally important goal, is to bridge the phenotype and genotype information characterising the diseases, in order to build a comprehensive body of knowledge from the existing and emerging patient reports. Consequently, the three important pillars of the domain, as depicted in Figure 1, have been mapped to the top level classes of the ontology, i.e., **Bone Dysplasia**, **Gene Mutation**, **Gene **and **Phenotypic Composite**. The phenotype information is also captured by adopting concepts from external ontologies.

In order to avoid ambiguous interpretation and to enable compatibility between Bone Dysplasia Ontology concepts and concepts from other ontologies, the top level classes are rooted in entities defined by the Basic Formal Ontology (BFO, http://www.ifomis.org/bfo[13], and where possible by the Ontology of General Medical Science (OGMS) [14]--a middle ontology rooted in BFO, which provides a specific framework for medicine, to be extended by specialised ontologies. The concept mappings are listed in the following: (i) **Bone Dysplasia **represents a **ogms:OGMS_0000047 **(*Genetic disorder*), (ii) **Gene **is a **snap:MaterialEntity**; (iii) **Gene Mutation **represents **snap:SpecificallyDependentContinuant**(s) as every gene mutation is specific to a particular gene; (iv) **Phenotypic Composite **represents a **ogms:OGMS_0000023 **(*Phenotype*).

The **Bone Dysplasia**, **Gene **and **Protein **terms were manually extracted from the 2010 Revision of the ISDS Nosology [2]. **Gene **classes were also augmented with references to external resources, such as MeSH http://www.nlm.nih.gov/mesh/, OMIM or Uniprot. **Gene Mutation **descriptions were designed according to the Mutation Nomenclature of the Human Genome Variation Society [15], to capture the offset of the mutation and the original and mutated content. For example, **GLY380ARG, 1138 G-A **has a **NCI: Missense Mutation **type attached, an *offset *of 1138, *count *1, *original content *G and *mutated content *A.

**Figure 2:**
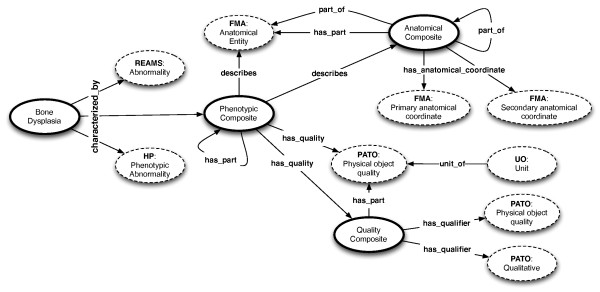
**Connecting the bone dysplasias to phenotype information**. Dotted circles represent concepts from external ontologies. The direction of the arrows have the same meaning as in Figure 3.

In recent years, phenotype ontologies have been seen as an invaluable source of information, which can enrich and advance evolutionary and genetic databases [16]. One of the pioneering example, and currently the most comprehensive source of such information is the Human Phenotype Ontology (HP) [17]. We imported concepts from HP to augment the intrinsic skeletal dysplasia genetic information with phenotypic descriptions. However, as noted by [18], most of the terms in HP implicitly combine anatomical entities with qualities. For example, *Mitral valve prolapse *(**HP:HP_0001634**) can be decomposed into the anatomical entity *Mitral valve *and the quality *prolapsed*. As a result, in order to capture information currently not covered by HP, but by also taking into account the aforementioned distinction, our top level concept **Phenotypic Composite **enables the composition of an **Anatomical entity**, concept imported from the Foundational Model of Anatomy Ontology [19] or an **Anatomical Composite**, concept we introduce to model partonomies of anatomical entities, and a **Physical Object Quality**, concept imported the Phenotype and Trait Ontology (PATO) [18] or a **Quality Composite**, a concept we define to capture conjunctions of qualities and qualifiers (e.g., *mildly bowed*). The complete structure of the **Phenotypic Composite **can be seen in Figure 2. Qualities may also have measurement units attached via concepts imported from Units of Measurement ontology (UO, http://purl.org/obo/owl/UO). Finally, additional phenotypic information, with an accent on clinical radiographic features, has been foreseen via the import of the **Abnormality **concept of the Dynamic Radiological Electronic Atlas of Malformation Syndromes ontology (dREAMS, http://d-reams.org/?page_id=84).

The current import of all external concepts followed the minimum information to reference an external ontology term (MIREOT) guidelines [20]. The platform sustaining the evolution of the ontology will ensure that the import of any additional external concepts will respect the same guidelines.

The structure of the ontology is a directed acyclic graph (DAG) based on taxonomical relations and using the *rdfs:subClassOf *construct. All classes have fully qualified URIs, while the human-readable description is provided via the *rdfs:label *property. Alternative definitions (e.g., synonyms or acronyms), in addition to references to external entities are defined by existing or custom OWL annotation properties, such as *skos:altLabel*, *chromosomal_locus*, *uniprot_id*, *omim_no *or *mesh_id*. The metadata describing the ontology is represented using the DublinCore vocabulary http://purl.org/dc/terms/ and its defined properties: *dc:title, dc:creator, dc:contributor*and *dc:publisher*.

We have formalised the ontology using OWL-DL [21], one of the three sub-languages of the Web Ontology Language (OWL) because it provides a maximum expressiveness without losing computational completeness. OWL-DL defines constructs that enable: (i) boolean combinations of class expressions (such as union or intersection, required to integrate diverse vocabularies for describing the phenotype information); (ii) as well as disjointness and equivalence class axioms; and (iii) arbitrary cardinality restrictions. Furthermore, the sublanguage has also developed a wide range of mature reasoners, which makes it an ideal candidate for real-world practical applications.

From a pragmatical perspective, we opted for using a logical formalism, because only a well-structured, logical representation framework is able to encode the relations existing between phenotypic and genotypic characteristics in the context of particular bone dysplasias. The resulting class axioms not only encode properly the conceptual real-world knowledge of the domain (e.g., **Achondoplasia **is characterized by a mutation in gene **FGFR3 **and by **Hydrocephalus **or **Lumbar hyperlordosis**), but also enable us to use this conceptual knowledge to perform reasoning on patient instance data.

The Bone Dysplasia Ontology was curated manually using the Stanford Protege-OWL 4.1 http://protege.stanford.edu/ ontology editor. For reasoning purposes, the ontology imports (via *owl:imports *statements) the Human Phenotype Ontology and the Phenotype and Trait Ontology, but also specific concepts from different other ontologies, as specified above and further described in the following section. The consistency checking has been performed by running the OWL-DL Pellet v2.1.2 [22] and Hermit v1.3.3 [23] reasoners over the ontology, to analyse both the class and object property definitions.

## Results and discussion

This section details the classes defined by the Bone Dysplasia Ontology and the class axioms and relations that we have introduced in order to accurately model the existing knowledge in the domain. It also discusses the availability of the ontology and our envisioned revision and extension cycle.

### The Bone Dysplasia Ontology classes

The structure of the ontology is conceptually built around three main knowledge pillars: bone dysplasias, genotype information and phenotype information, as depicted in Figure 1. The ontology consists of 1228 own-defined classes, of which 515 define bone dysplasias, 254 define genes, 361 define gene mutations and 224 define proteins.

**Figure 4:**
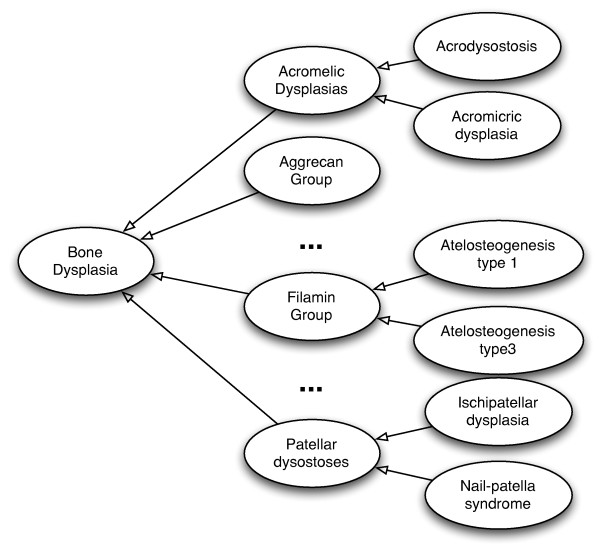
**Bone Dysplasias classification**. The main **Bone Dysplasia **concept is connected to lower level concepts via rdfs:subClassOf relations.

The skeletal dysplasias component comprises the hierarchy of diseases, starting from the **Bone Dysplasia **super-concept which is refined via taxonomical relations (i.e., *rdfs:subClassOf*) to 40 specific groups of diseases (e.g., **rotect Acromelic Dysplasias**, **Aggrecan Group **or **Patellar dysostoses**) and then to dysplasias defined within the groups. Figure 4 presents a small portion of the classification. In principle, the hierarchy has two levels, i.e., the group level and the leaf level representing bone dysplasias, however, there are also cases where the depth of the hierarchy is three. Such an example exists in the **Craniosynostosis syndromes **group, where the class **Pfeiffer syndrome FGFR2-related **has two subclasses **Antley-Bixler variants caused by FGFR2 mutations **and **Jackson-Weiss syndrome**. In principle, all classes defined at this level, such as the aforementioned two, represent diseases maintained only for historical purposes. The Nosology mentions them as being subsumed by some other disorders (via simple observations), like the **Pfeiffer syndrome FGFR2-related **in our example, and hence we added them as subclasses of the corresponding concepts in the hierarchy.

Similar or equivalent bone dysplasia concepts, including a **HP_0002652 **(*Skeletal dysplasia*) super-concept, are also defined by the Human Phenotype Ontology. However, a correct alignment between these terms and the terms defined within our ontology, both from the domain and the logical perspectives, cannot be realised due to either the vagueness or the improper granularity of the concepts. For example, HP defines concepts such as **HP_0005716 **(*Lethal skeletaldysplasia*) or **HP_0005685 **(*Severe skeletal dysplasia*), which seem to be rather qualities than proper disease definitions. Similarly, concepts like **HP_0002654 **(*Multiple epiphyseal dysplasia*), are defined in our ontology at a much more fine-grained level via several concepts, e.g., in this case via seven classes (see **Multiple epiphyseal dysplasia and pseudoachondroplasia Group**).

**Figure 3:**
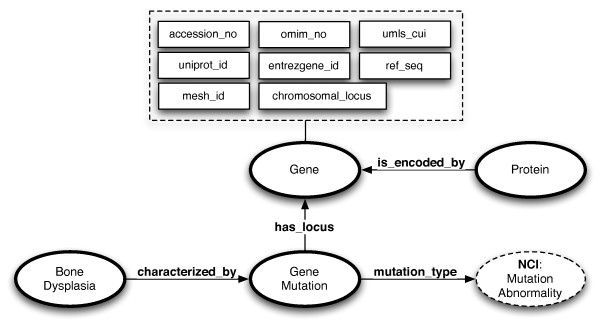
**Connecting the bone dysplasias to genotype information**. Dotted circles represent concepts from external ontologies. The dotted box represents annotation properties attached to the **Gene **concept. The direction of the arrows shows domain-range association in the property definition.

The genotype information pillar captures **Gene Mutation**(s) and their associated **Gene**(s) and **Proteins **(see Figure 3). Each of these concepts have a corresponding class in the ontology and subsume particular sub-concepts. **Gene Mutation **classes are related to **Gene **classes via the *has_locus *relation. Similarly, **Protein **classes are related to **Gene **classes via the *is_encoded_by *relation. The naming of the subclasses of these three concepts follows an incrementally encoded structure, e.g., **GM0000001 **for a gene mutation, **G0000001 **for a gene, and **P0000001 **for a protein. However, genes and proteinsalso have human readable names provided via the *rdfs:label *property and synonyms via the *skos:altLabel *property. For example, **G0000047 **has the label *MNX1 *and the alternative label (or synonym) *HLXB9*.

Gene mutations are defined according to the Mutation Nomenclature of the Human Genome Variation Society [15], to capture the offset of the mutation, along with the original and mutated content, via four datatype properties: *original_content*, *offset*, *count *and *mutated_content*. The type of the mutation is signalled by the *mutation_type *relation between **Gene Mutation **and **NCI:Mutation Abnormality**, the latter concept being imported in our ontology together with its entire sub-structure (see below for a gene mutation example). **Gene **concepts are linked to multiple external resources, e.g., OMIM, Uniprot or MeSH via corresponding annotation properties: *omim_no*, *uniprot_id*, *mesh_id*, *umls_cui*, *ref_seq*, *entrezgene_id *and *accesion_no*.

As a side remark, the ontology contains a second **Gene Mutation **class, imported from the NCI thesaurus as part of the **NCI:Mutation Abnormality **sub-tree. The BDO and the NCI **Gene Mutation **classes are not equivalent. The BDO **Gene Mutation **is an entity that describes actual gene mutations (linked to a **Gene **and having a type, encoding, offset, etc.). The NCI **Gene Mutation **is, in reality, improperly defined because it refers to a type of mutation and not to a gene mutation per se. This can be easily observed by analysing the concepts imported from NCI under the **NCI:Mutation Abnormality **super-concept, which describe different types of gene mutations. However, since we rely on these mutation type concepts and import them according to the MIREOT principle, we were not able to omit this particular concept, and hence to avoid confusion.

The phenotype information (depicted in Figure 2) is recorded in a highly extensible manner via the main class **Phenotypic Composite**. The complex nature of skeletal dysplasias can be observed in particular in the wide range of clinical and radiographic characteristics manifested by patients. Consequently, we opted for re-using concepts from known ontologies that subsume most of the possibly arising phenotype information in patient records, e.g., **REAMS:Abnormality **for radiographic features and **HP:HP_0000118 **(*Phenotypic abnormality*) for other phenotypic findings. As discussed earlier, our **Phenotypic Composite **class represents a composite element that connects conceptually an **otect FMA:Anatomical entity **or an **Anatomical Composite **to a **PATO:Physical Object Quality **or a **Quality Composite **using the *describes *and *has_quality *relations, or can build upon existing composites via the OBO *has_part *relation in addition to connecting a **PATO:Physical Object Quality **or a **Quality Composite **via the *has_quality *relation. The **FMA:Anatomical entity **and **PATO:Physical Object Quality **concepts have been imported in our ontology, however, choosing particular sub-concepts and specialising their relations to particular dysplasias is deferred to the community and supported by the platform described later in the paper. Hence, **Phenotypic Composite **carries a scaffolding role onto which particular elements can be created to complement the gaps in the current phenotype ontologies. Some definition examples are, however, presented both in the ontology, as well as in the following section.

### Class axioms and relationships

Table 1 lists the main relations introduced by the Bone Dysplasia Ontology. The *mode_of_inheritance *relationship links **Bone Dysplasia**(s) to diverse modes of inheritance from the Human Phenotype Ontology (via **HP_0000005**). The *mutation_type *relation provides a connection between **Gene Mutation **and the **NCI:Mutation Abnormality **that defines all possible gene mutation types, while the *has_locus *relation links **Gene Mutation **to a **Gene**. Finally, the *characterized_by *relation provides support for associating **Bone Dysplasia**(s) to **Phenotypic Composite**(s), **Gene Mutation**(s) or HP:HP_0000118 (Phenotypic Abnormality). To these are added the two relations mentioned above, i.e., *describes *and *has_quality*, and the *has_anatomical_coordinate *relation, used to connect a **Anatomical Composite **to a **FMA:Primary anatomical coordinate **or **FMA:Secondary anatomical coordinate**, and the *has_qualifier *relation that enables the attachment of qualifiers from **PATO:PATO_0000068 **(*Qualitative*) and **protect PATO:PATO_0001241 **(*Physical object quality*) to **Quality Composite**(s).

**Table 1:** Relations defined in the Bone Dysplasia ontology

Relation	Domain	Range
*characterized_by*	Bone Dysplasia	Phenotypic Composite, Gene Mutation
		HP:HP_0000118 (*Phenotypic abnormality*)
*mode_of_inheritance*	Bone Dysplasia	HP: HP_0000005 (*Mode of inheritance*)
*has_locus*	Gene Mutation	Gene
*mutation_type*	Gene Mutation	NCI:Mutation Abnormality
*is_encoded_by*	Protein	Gene
*describes*	Phenotypic Composite	Anatomical Composite, FMA:Anatomical_Entity
*has_quality*	Phenotypic Composite	Quality Composite,
		PATO:PATO_0001241(*Physical object quality*)
*has_qualifier*	Quality Composite	PATO:PATO_0000068(*Qualitative*),
		PATO:PATO_0001241(*Physical object quality*)
*has_anatomical_coordinate*	Anatomical Composite	FMA:Primary_anatomical_coordinate,
		FMA:Secondary_anatomical_coordinate

A major aim of the Bone Dysplasia ontology is to underpin a community-driven knowledge curation platform that enables collaborative decision making and knowledge exchange among the experts in the field. In order to support the decision making process (i.e., collaborative diagnosis), as well as the transfer of knowledge from particular patient studies to the generic concept definitions, we encoded the semantics of the emerging knowledge discoveries in class axioms and restrictions. Furthermore, to reflect the current domain knowledge about each specific dysplasia accurately, these class axioms are specialised at the lower levels of the **Bone Dysplasia **concept with more specific details. As a result, more than 70% of actual bone dysplasia concepts are linked to gene mutations, and around 80% of the same concepts have phenotype information attached (via more than 2,000 phenotypes imported from the Human Phenotype Ontology). The lack of class axioms in the rest of the bone dysplasia concepts is due, in principle, to two factors. From the genetic perspective, the corresponding bone dysplasias currently have no established links with particular genes, while from the phenotype perspective, we were, until now, unable to mine disorder--phenotype relations for the corresponding bone dysplasias.

The class definition of three of the top-level concepts (as **Gene **is an independent material entity) are presented below, using the OWL Manchester syntax:

Class: Bone_Dysplasia

SubClassOf:

OGMS:OGMS_0000047

SubClassOf:

characterized_by only (REAMS:Abnormality or HP:HP_0000118

or Phenotypic_Composite or Gene_Mutation)

SubClassOf:

mode_of_inheritance only HP:HP_0000005

Annotations:

skos:description "A genetic disorder that involves abnormal

development of bones and connective tissues."

*Definition: ***Bone_Dysplasia **is defined as a specialisation is defined as a specialisation has two restrictions: (i) all concepts that characterise this entity (via *characterized_by*) are **Gene_Mutation**s or **Phenotypic_Composite**s, or **REAMS:Abnormality **or **HP:HP_0000118 **(*Phenotypic abnormality*), and (ii) all concepts providing a *mode_of_inheritance *for this entity are **HP:HP_0000005 **(*Mode of inheritance*).

Class: Gene_Mutation

SubClassOf:

SNAP:SpecificallyDependentContinuant

SubClassOf:

has_locus only Gene and has_locus some Gene

SubClassOf:

mutation_type only NCI:Mutation_Abnormality

and mutation_type some NCI:Mutation_Abnormality

Annotations:

skos:description "A change or alteration in a gene."

*Definition: ***Gene_Mutation **is defined as a specialisation of an entity that has two restrictions: (i) all concepts acting as a locus for this entity (via *has_locus*) are **Gene**s and there is at least one such **Gene **that is the locus of this entity, and (ii) all concepts that define the *mutation_type *for this entity are **NCI:Mutation_Abnormality **and there is at least one such **NCI:Mutation_Abnormality **that provides a mutation type.

Class: Phenotypic_Composite

SubClassOf:

OGMS:OGMS_0000023

SubClassOf:

(has_part some Phenotypic_Composite

and has_part only Phenotypic_Composite)

or

(describes some FMA:Anatomical_entity

and describes only FMA:Anatomical_entity)

or

(describes some Anatomical_composite

and describes only Anatomical_composite)

SubClassOf:

(has_quality only PATO_0001241

and has_quality some PATO_0001241)

or

(has_quality only Quality_Composite

and has_quality some Quality_Composite)

Annotations:

skos:description "A continuant describing the conjunction

between a quality and an anatomical part or an anatomical

composite."

*Definition: ***Phenotypic_Composite **is defined as a specialisation of an entity that has two restrictions: (i) the entity either *has_part *some concepts that are all **Phenotypic_Composite **and there exist at least one such **Phenotypic_Composite **that is a part of the entity, or all concepts described by this entity (via *describes*) are **FMA:Anatomical_entity **and there is at least one such **FMA:Anatomical_entity **that is described by the entity, or all concepts described by this entity (via *describes*) are **Anatomical_Composite **and there is at least one such **Anatomical_Composite **that is described by the entity, and (ii) all concepts that define a quality for this entity (via *has_quality*) are **PATO:PATO_0001241 **and there is at least one such **PATO:PATO_0001241 **that provides a quality, or all concepts that define a quality forthis entity (via *has_quality*) are **Quality_Composite **and there is at least one such **Quality_Composite **that provides a quality.

Below, we illustrate a series of concrete concept definition examples, for the **Achondroplasia **and **GM0000001 **classes and two particular **Phenotypic Composite**s--*Translucency of proximal femur *and *Oval translucency of proximal femur*, by showing the some of the definition constraints, and in the case of the gene mutation, the information captures along the lines of the Mutation Nomenclature of the Human Genome Variation Society:

Class: Achondroplasia

SubClassOf:

characterized_by only (GM000001 or GM000361

or HP_0000238 or HP_0002938 or HP_0002968 or HP_0003505 or ...)

SubClassOf:

mode_of_inheritance only HP_0000006 and

mode_of_inheritance some HP_0000006

Class: GM000001

SubClassOf:

has_locus only G0000001 and has_locus some G0000001

SubClassOf:

mutation_type only NCI:Missense_Mutation and

mutation_type some NCI:Missense_Mutation

Annotations:

encoding "GLY380ARG, 1138 G-A", offset 1138,

original_content "G", mutated_content "A"

Class: PC_0000004

SubClassOf:

describes only AC_0000001 and describes some AC_0000001

SubClassOf:

has_quality only PATO:PATO_0001354 and1

has_quality some PATO:PATO_0001354

Annotations:

label "Translucency of proximal femur"

skos:description "Translucent proximal area of femur"

Class: AC_0000001

SubClassOf:

has_part only FMA:Femur and has_part some FMA:Femur

SubClassOf:

has_anatomical_coordinate only FMA:Proximal and

has_anatomical_coordinate some FMA:Proximal

Annotations:

label "Proximal femur"

skos:description "The proximal area of the femur"

Class: PC_0000005

SubClassOf:

describes only AC_0000001 and describes some AC_0000001

SubClassOf:

has_quality only QC_0000001 and has_quality some QC_0000001

Annotations:

label "Oval translucency of proximal femur"

skos:description "Oval-shaped translucent area of the proximal femur"

Class: QC_0000001

SubClassOf:

has_part only PATO:PATO_0001354 and

has_part some PATO:PATO_0001354

SubClassOf:

has_qualifier only PATO:PATO_0000947 and

has_qualifier some PATO:PATO_0000947

Annotations:

label "Oval translucency"

skos:description "Oval-shaped area of translucency"

### Availability

Table 2 summarises the main characteristics of the Bone Dysplasia Ontology. The current release of the ontology has the version number 1.5, and the namespace of the ontology is http://purl.org/skeletome/bonedysplasia. The classification of the bone dysplasias defined in the ontology corresponds to the ISDS Nosology 2010 [2], which has only recently been published. The ontology can be retrieved directly from the given namespace, or visualised using the NCBO BioPortal at: http://bioportal.bioontology.org/ontologies/1613.

**Table 2:** Bone Dysplasia Ontology fact sheet

Name	Bone Dysplasia Ontology
Namespace	http://purl.org/skeletome/bonedysplasia
Prefix	BDO
Scope	skeletal dysplasias, genes, proteins,
	gene mutations and phenotypic
	characteristics in human
Format	OWL-DL
Number of classes	1228
Dependencies (import)	HP, PATO, NCI (*Gene mutation types*)
Dependencies (weak)	FMA, REAMS
Annotations	*rdfs:label, skos:altLabel, uniprot_id* *entrezgene_id, ref_seq, mesh_id, locus,* *omim_no, umls_cui, accession_no* *skos:description*

The design of the ontology aims to re-use and adopt existing vocabularies in order to minimize the re-invention, duplication and overlap of concepts. Consequently, the ontology imports, following the MIREOT guidelines [20], a series of concepts from external resources, as previously discussed. Additionally, the **Gene **concepts include references to OMIM, Uniprot, MeSH, and UMLS via corresponding annotation properties, while the **Bone Dysplasia **concepts refer to OMIM and MeSH.

### Revising and extending the Bone Dysplasia Ontology

The Bone Dysplasia ontology has been built as a foundation block for SKELETOME--the skeletal dysplasia knowledge curation platform (described in the following section). As such, support for extensibility is important, to cope with the complex and evolving nature of the field. Consequently the SKELETOME platform has been designed to enable roundtrip knowledge engineering, which assumes the evolution of the ontology. New discoveries emerging from patient studies will be easily transferred at the conceptual level by domain experts (via class axioms) through extensions to the ontology and through additional semantic inference rules, as well as at the instance level as new case data becomes available. In addition to refined class definitions via specialised restrictions, the platform allows users with editorial roles to alter the bone dysplasia classification, by creating or deleting groups, or by moving diseases between groups. This leads to a continuous evolution of the ontology and inherently of the Nosology and bone dysplasia knowledge.

### Comparison to related ontological resources

Among the three pillars of the Bone Dysplasia ontology, the actual skeletal dysplasia knowledge (representing the core of the ontology) is covered only superficially in other ontologies and vocabularies. Examples such as the Systematized Nomenclature of Medicine--Clinical Terms (SNOMED-CT, http://www.ihtsdo.org) [24], REAMS, the NCI Thesaurus [25] or the Human Disease Ontology include, as also highlighted in the Background section, only high level concepts denoting the most commonly known dysplasias. None of these existing related vocabularies attempt to capture related genotype or phenotype information. The added value of the Bone Dysplasia ontology stands in the comprehensive classification of these disorders, in addition to an accurate descriptions (via class axioms and relations) of their main genetic and phenotypic characteristics. We regard the other ontologies, in particular REAMS, SNOMED and the NCI thesaurus, as effective complements and important resources to be cross-referenced and re-used (to avoid redundancy) to describe the phenotype and genotype information of bone dysplasias.

To date, the integrity of the ontology has been ensured by the domain experts-driven curation. Future testing of its applicability will be evidenced by the extent of its changes over time and the future growth of the SKELETOME knowledge base and its associated community of users.

## Community-driven knowledge curation

The increasing use of ontologies in Healthcare and Life Sciences has led to novel ways of processing digital content, which in turn have introduced new possibilities of dealing with scientific publications and data [6]. Such content processing techniques make knowledge more open and exploitable than ever before [8,26].

Our focus is on ensuring a continuous enrichment and evolution of the ontology by transferring knowledge present in existing and emerging patient case studies into class axioms or cross-references to external phenotype ontology concepts. In order to achieve this, we developed the SKELETOME platform http://skeletome.metadata.net/skeletome, a community-driven knowledge curation platform that enables collaborative input, sharing and re-use of data and information among experts in the skeletal dysplasia domain. SKELETOME provides a central access point to a rich skeletal dysplasia knowledge base, supported by low-level features, such as user and group-based access and privacy control. At the same time, from a high-level perspective, the anonymised pool of case studies enables statistical inference for knowledge discovery purposes or computer-assisted diagnosis.

SKELETOME is built as a Drupal 7 http://drupal.org/ instance, thus inherently providing the collaborative aspects, and also allowing us to develop custom modules to suit our needs. The Bone Dysplasia Ontology acts as the knowledge back-bone of the platform. Each of the disease concepts present in the hierarchy of skeletal dysplasias has been imported, via its own module, into the platform and has an associated human-readable page. The system structure is similar to a knowledge Wiki that is built around the ontology. The user-friendly page corresponding to each dysplasia, presents a summary of the dysplasia and contains pointers to external references. Registered members of the community can add facts grounded in scientific publications (similar to the OMIM structure) and can discuss facts added by other members. Members with editorial role have the ability of editing the summary of dysplasias by incorporating the facts widely accepted by the community. They are also able to alter the bone dysplasia pages by, for example, moving them between groups. Such operations have a direct impact on the ontology and are immediately reflected in the underlying knowledge base. The continuous logical correctness of the ontology is always enforced by the platform, without the experts noticing it. In practice, we have created a round-trip knowledge engineering process, driven by the experts in the community (only a few experts have editorial roles) who are not required to possess any ontology engineering skills.

**Figure 5:**
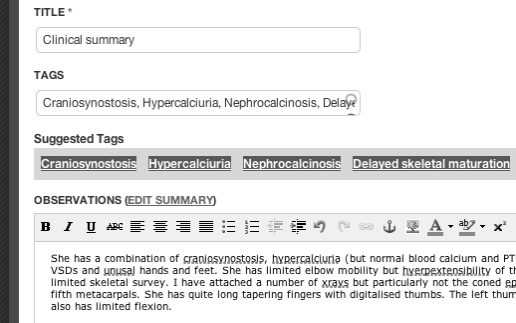
**Semantic Annotation in SKELETOME**. Tagging clinical summaries in SKELETOME using the Bone Dysplasia ontology and references to external ontologies. The example shows a clinical summary tagged with terms from the Human Phenotype Ontology and subclasses of the **Bone Dysplasia **concept.

**Figure 6:**
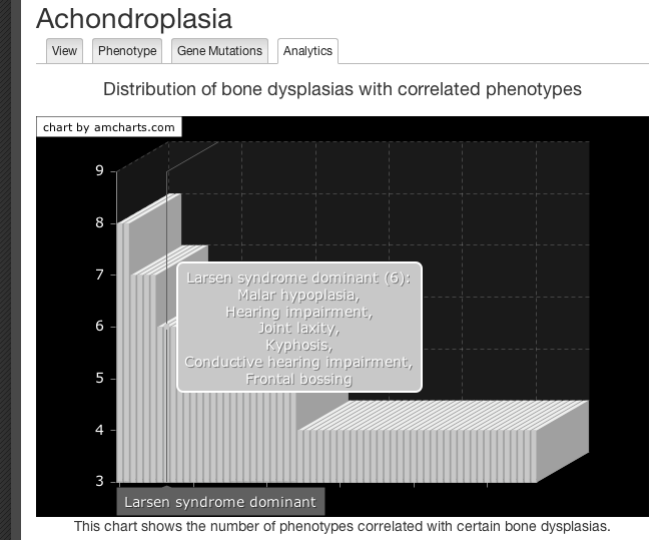
**Ontology analytics for data exploration**. The SKELETOME platform provides facilities for performing ontology analytics, presented to the user in form of charts for data exploration purposes. The chart in the figure depicts the distribution of bone dysplasias with correlated phenotypes, in the context of the **Achondroplasia **concept. The chart cursor shows the six overlapping phenotypes with **Larsen syndrome dominant**.

The development of the actual knowledge-base about bone dysplasias is supported by SKELETOME's knowledge engineering cycle. On one side, the BDO concepts (including also concepts from the imported ontologies) are used to annotate patient case studies that can be uploaded, analysed and discussed by the members of the community. More concretely, the platform enables manual and automatic semantic annotation of clinical summaries (see Figure 5), as well as manual annotation of X-Ray imagery. In addition to annotation, SKELETOME uses the ontology to provide support in the collaborative diagnosis process via an underlying decision support mechanism, that computes probabilistic correlations of phenotypes in the context of a particular disorder, or raked list of disorders given particular phenotypes. The actual mechanisms perform association rule mining on existing patient data and refines the resulting rules based on the class axioms of the corresponding disorders before computing the final probabilistic rankings. Overall, the patient information is automatically linked, via the underlying ontological concepts, to the bone dysplasia concepts and pages. On the other side, from the dysplasias perspective, the ontology creates an integrated view on the phenotype and genotype emerging from patient reports and evolves based on the findings provided by the analysis of patient cases combined with the current domain knowledge. This is presented to the user in form of a ontology analytics service for exploratory purposes and is realised via direct querying on the ontology (see Figure 6 for an example).

The SKELETOME platform and knowledge-base, underpinned by the Bone Dysplasia ontology, represents an ideal approach by which experts in the skeletal dysplasias domain can collaboratively document, expand and maintain a curated body of the knowledge which will lead to accelerated innovation and scientific breakthroughs in their field.

## Conclusions

The Bone Dysplasia ontology described in this paper, represents the most comprehensive structured knowledge source for the skeletal dysplasias domain. It provides the means for integrating and annotating clinical and research data, not only at the generic domain knowledge level, but also at the level of individual patient case studies--by enabling links between individual cases and publicly available genotype and phenotype resources. The community-driven curation process ensures a shared conceptualisation of the domain knowledge and its continuous incremental evolution. Future development of both the ontology and the SKELETOME platform will focus on advancing the reasoning and knowledge extraction services--which will hopefully lead to the discovery of previously unknown relationships between gene mutations, phenotype characteristics and bone dysplasias and the discovery of new drugs to combat disorders associated with human bone and cartilage development.

## Competing interests

The authors declare that they have no competing interests.

## Authors' contributions

JH and AZ formulated the basic idea behind SKELETOME. JH coordinates the project. TG leads the development of the project. TG, AZ and JH designed the ontology. TG developed the ontology and wrote the paper. AZ provided the domain expertise behind the class axioms. All authors read and approved the final manuscript.
